# Bolstering fitness via CO_2_ fixation and organic carbon uptake: mixotrophs in modern groundwater

**DOI:** 10.1038/s41396-021-01163-x

**Published:** 2021-12-07

**Authors:** Martin Taubert, Will A. Overholt, Beatrix M. Heinze, Georgette Azemtsop Matanfack, Rola Houhou, Nico Jehmlich, Martin von Bergen, Petra Rösch, Jürgen Popp, Kirsten Küsel

**Affiliations:** 1 Aquatic Geomicrobiology, Institute of Biodiversity, Friedrich Schiller University Jena, Dornburger Strasse 159, 07743 Jena, Germany; 2 Institute of Physical Chemistry and Abbe Center of Photonics, Friedrich Schiller University Jena, Helmholtzweg 4, 07743 Jena, Germany; 3 Leibniz-Institute of Photonic Technology, Albert-Einstein-Strasse 9, 07745 Jena, Germany; 4 Department of Molecular Systems Biology, Helmholtz Centre for Environmental Research – UFZ, Permoserstrasse 15, 04318 Leipzig, Germany; 5 Faculty of Biosciences, Pharmacy and Psychology, Institute of Biochemistry, University of Leipzig, Brüderstrasse 32, 04103 Leipzig, Germany; 6 German Centre for Integrative Biodiversity Research (iDiv) Halle-Jena-Leipzig, Puschstrasse 4, 04103 Leipzig, Germany

**Keywords:** Water microbiology, Microbial ecology

## Abstract

Current understanding of organic carbon inputs into ecosystems lacking photosynthetic primary production is predicated on data and inferences derived almost entirely from metagenomic analyses. The elevated abundances of putative chemolithoautotrophs in groundwaters suggest that dark CO_2_ fixation is an integral component of subsurface trophic webs. To understand the impact of autotrophically fixed carbon, the flux of CO_2_-derived carbon through various populations of subsurface microbiota must first be resolved, both quantitatively and temporally. Here we implement novel Stable Isotope Cluster Analysis to render a time-resolved and quantitative evaluation of ^13^CO_2_-derived carbon flow through a groundwater community in microcosms stimulated with reduced sulfur compounds. We demonstrate that mixotrophs, not strict autotrophs, were the most abundant active organisms in groundwater microcosms. Species of *Hydrogenophaga*, *Polaromonas*, *Dechloromonas*, and other metabolically versatile mixotrophs drove the production and remineralization of organic carbon. Their activity facilitated the replacement of 43% and 80% of total microbial carbon stores in the groundwater microcosms with ^13^C in just 21 and 70 days, respectively. The mixotrophs employed different strategies for satisfying their carbon requirements by balancing CO_2_ fixation and uptake of available organic compounds. These different strategies might provide fitness under nutrient-limited conditions, explaining the great abundances of mixotrophs in other oligotrophic habitats, such as the upper ocean and boreal lakes.

## Introduction

From soils to deep-sea sediments, the vast majority of cells on Earth must find a way to thrive in environments devoid of photosynthesis [1]. To truly appreciate the global carbon cycle, it is important to understand the extent to which various types of cells rely upon allochthonous or autochthonous carbon input. The dependence on these carbon sources results in organisms occupying different niches in which they prefer heterotrophic or chemolithoautotrophic lifestyles, providing the foundation upon which trophic webs linking the entire subsurface biome are structured. Accurately gauging carbon fluxes and identifying active key players in these habitats is remarkably challenging despite the invaluable utility afforded by metagenomics to shed light on the metabolic capabilities of thousands of the organisms present [2–5].

Modern groundwater, i.e., water having ingressed into the subsurface within the past 50 years [6], is a transitionary ecosystem that connects surface habitats characterized by recently photosynthetically fixed carbon with the subsurface, which is devoid of this carbon source entirely [7–9]. In modern groundwater, inorganic electron donors such as reduced nitrogen, iron, and sulfur fuel chemolithoautotrophic activity, which takes over the role phototrophic primary production has at the surface [9–12]. Metagenomic-based studies have elucidated a diverse array of microorganisms bearing the metabolic potential for chemolithoautotrophy [4, 13–16], accounting for 12% to 47% of the microbial community detected in groundwater [17–20]. Discoveries such as these have cast doubt on paradigms portraying modern groundwater as being primarily inhabited by heterotrophic microbes fueled by organic material from the surface. We hypothesize that chemolithoautotrophic production of biomass dictates the carbon fluxes in the modern groundwater microbiome.

To validate this hypothesis, we implemented a novel approach, Stable Isotope Cluster Analysis (SIsCA), to render a time-resolved, quantitative assessment of CO_2_-derived carbon flow through the groundwater food web. By coupling stable isotope probing (SIP) with genome-resolved metaproteomics [21, 22], we leveraged the high sensitivity of Orbitrap mass spectrometry in SIP-metaproteomics to acquire exceedingly accurate quantitative data on ^13^C incorporation [23, 24]. SIsCA then employs a dimensionality reduction approach to visualize the acquired data from the ^13^C-SIP time-series experiment and identify clusters of microbes with similar carbon assimilation profiles—allowing us to ultimately discern trophic interactions between individual members of the microbial community.

To examine the role of chemolithoautotrophy in the groundwater microbiome, we amended groundwater microcosms with ^13^CO_2_. Thiosulfate was used as an electron donor, as it is regularly released into groundwater via rock weathering [25–28]. Although organisms bearing the genetic potential to oxidize reduced sulfur compounds are widespread in groundwater, their carbon acquisition preferences and the range of compounds they use are unclear [15, 16, 29]. A previous denitrifying thiosulfate enrichment culture from groundwater of the Hainich Critical Zone Exploratory (CZE) contained 75–95% of putative chemolithoautotrophs [30]. Under conditions favoring lithotrophic growth, we thus expected chemolithoautotrophy to be the primary source of organic carbon and a unidirectional carbon flux from autotrophs to heterotrophs. By mapping the quantitative information derived from SIsCA to metagenome-assembled genomes (MAGs), we were able to characterize carbon utilization and trophic interactions between active autotrophs and heterotrophs in the groundwater microbiome over a period of 70 days. High-resolution monitoring of carbon cycling and taxon-specific activities demonstrated that metabolically versatile mixotrophs, not strict autotrophs, drove the carbon flux in the groundwater microcosms, supplying up to 80% of the entire microbial carbon. Our work offers insights into groundwater carbon acquisition strategies and suggests that metabolically flexible mixotrophic lifestyles are optimal for microorganisms to flourish in such oligotrophic systems.

## Materials and methods

### Groundwater sampling and microcosms setup

Groundwater was collected from Hainich CZE well H41 (51.1150842N, 10.4479713E) in June 2018. Details on the geological and hydrochemical conditions in this well can be found in Supplementary Information. A total of 120 L of groundwater was sampled using a submersible pump (Grundfos MP1, Grundfos, Bjerringbro, Denmark). To collect biomass from the groundwater, 5 L fractions were filtered through each of twenty 0.2 µm Supor filters (Pall Corporation, Port Washington, NY, USA). The natural background of inorganic carbon (IC) in the groundwater was then replaced with defined concentrations of ^12^C or ^13^C. Two 3 L volumes of filtered groundwater were acidified to pH 4 with hydrochloric acid in 5 L bottles to eliminate any bicarbonate. Following that, ^12^C- or ^13^C-bicarbonate was dissolved in the groundwater to a final concentration of 400 mg L^−1^. Based on the molecular mass of bicarbonate of 61.02 g mol^−1^ and of carbon of 12.01 g mol^−1^, this corresponds to 79 mg C L^−1^, which is close to the in situ concentration [27]. The pH of groundwater samples was then adjusted to 7.2 by addition of ^12^C- or ^13^C-CO_2_.

Eighteen distinct microcosms were initiated for the ^13^C-SIP experiment. For each microcosm, one sample-laden 0.2 µm filter was placed into a 500 mL bottle containing 300 mL of treated groundwater (as described above). Nine bottles were sourced with water containing ^12^C-bicarbonate and the other nine with water containing ^13^C-bicarbonate. Two additional bottles were prepared, each by transferring one 0.2 µm filter into a 1 L bottle containing 350 mL of untreated groundwater. One of these bottles was supplemented with 150 mL sterile D_2_O (final concentration 30%, *v* : *v*) and the other with 150 mL sterile milliQ H_2_O. Sodium thiosulfate and ammonium chloride were added to all bottles to a final concentration of 2.5 mM and 15 µM, respectively. Finally, all bottles were incubated with shaking (100 r.p.m.) at 15 °C in the dark. See Supplementary Information for details on hydrochemical analyses conducted during incubation.

### Detection of cellular activity by Raman microspectroscopy

Microcosms supplemented with D_2_O or H_2_O were sampled regularly during the first 7 weeks of incubation to quantify the incorporation of deuterium into the biomolecules of active cells (i.e., carbon-deuterium [C-D] bonds) via single-cell Raman microspectroscopy analysis. In preparation for Raman microspectroscopy, 1 mL of sample was pre-filtered through a 5 µm filter and then the cells contained in the filtrate were washed three times with milliQ H_2_O via centrifugation (10,000 × *g*, 2 min). Pellets were resuspended in 50 µL milliQ H_2_O and 10 µL of the final suspension was placed on nickel foil (Raman substrate) and allowed to air dry at room temperature. Microbial cells were located via dark field microscopy and measurements were collected using a Raman microscope (BioParticleExplorer 0.5, rap.ID Particle Systems GmbH) with an excitation wavelength of 532 nm (solid-state frequency-doubled Nd:YAG module [Cobolt Samba 25 mW]; laser power = 13 mW at sample). The laser was focused with an ×100 objective (Olympus MPLFLN 100xBD) across a lateral spot of <1 µm. Backscattered light (180°) was diffracted using a single-stage monochromator (Horiba Jobin Yvon HE 532) with a 920 line mm^−1^ grating. Spectra were then registered with a thermoelectrically cooled charge-coupled device camera (Andor DV401-BV), resulting in a resolution of ~8 cm^−1^. A 5 s integration period was applied per Raman spectrum (−57 to 3203 cm^−1^).

### Processing and analysis of Raman data

Processing and statistical analysis of raw Raman data were achieved with GNU R software [31]. Cosmic spikes were removed from the spectra [32]. A wavenumber calibration was then applied using 4-acetamidophenol standard spectra [33] and an intensity calibration was performed using the SRM2242 standard [34, 35]. The contribution of fluorescence was removed from spectra using the asymmetric least-squares baseline correction method [36]. Finally, spectra were vector-normalized and subjected to dimensionality reduction via principal component analysis (PCA). Five principal components were used to build a linear discriminant analysis classification model, which was applied to differentiate between deuterium-labeled and unlabeled bacterial cells. Deuterium uptake was expressed as the C-D ratio, i.e., A(C-D)/[A(C-D) + A(C-H)], which was calculated by integrating the areas of the C-H (2800–3100 cm^−1^) and C-D (2040–2300 cm^−1^) stretching vibration bands. Monitoring deuterium incorporation into microbial cells helped gauge metabolic activity, as well as determine optimal time points for sampling.

### Sampling and biomolecule extraction

After 21, 43, and 70 days of incubation, biomass was recovered from microcosms by filtering aqueous phases through 0.2 µm Supor filters (Pall Corporation). Filters used for pre-incubation biomass enrichment were combined with the filters used to remove the aqueous phases. A combined DNA and protein extraction was performed using a phenol/chloroform/isoamylalcohol-based protocol, as previously described [37]. Details regarding 16S rRNA gene amplicon sequencing and quantitative SIP of DNA are provided in Supplementary Information.

### Metagenomic analysis

Shotgun sequencing was performed on DNA samples selected from four ^12^C groundwater microcosms: 1 replicate each following 21 and 43 days of incubation and 2 replicates following 70 days of incubation. Samples were selected with the aim of covering greatest taxonomic diversity, as per the results of 16S rRNA gene amplicon sequencing data. DNA fragment sizing, quantification, integrity, and purity were determined using an Agilent 2100 Bioanalyzer (Santa Clara, CA, USA). Library preparation was achieved with a NEBNext Ultra II DNA Lib Prep Kit (New England Biolabs, Ipswich, MA, USA) in accordance with protocols provided by the manufacturer. Multiplexed sequencing in one flow cell of a NextSeq 500 system (300 cycles; Illumina, Inc., San Diego, CA, USA) ensued to generate 150 base paired-end reads. Details on generation of MAGs from sequencing data and on curated MAGs are provide in Supplementary Information and Table S1.

### Metaproteomics analysis

Proteins extracted from groundwater microcosms were first subjected to SDS polyacrylamide gel electrophoresis, followed by in-gel tryptic cleavage as previously described [37]. After reconstitution in 0.1% formic acid (*v* : *v*), liquid chromatography-tandem mass spectrometry (LC-MS/MS) analysis was performed in LC chip coupling mode on a Q Exactive HF instrument (Thermo Fisher Scientific, Waltham, MA, USA) equipped with a TriVersa NanoMate source (Advion Ltd, Ithaca, NY, USA). Raw data files were analyzed using the Sequest HT search algorithm in Proteome Discoverer (v1.4.1.14, Thermo Fisher Scientific, Waltham, MA, USA). To create a reference database for protein identification, genes of all binned contigs were called and annotated with Prokka v1.13.3 [38] using the --metagenome option and the Prokka database (2019_09) based on SwissProt. Gene sequences were translated to amino acid sequences, which were combined in one database, retaining the functional information from Prokka and the taxonomic classification from the dereplicated and refined MAGs (Dataset S1). The following parameters were applied for protein identification: enzyme specificity was set to trypsin, two missed cleavages were allowed, oxidation (methionine) and carbamidomethylation (cysteine) were selected as modifications, and peptide ion and Da MS/MS tolerances were set to 5 p.p.m. and 0.05, respectively. Peptides were considered identified upon scoring a *q*-value < 1% based on a decoy database and obtaining a peptide rank of 1. Only peptides unique to one protein sequence, unique to one MAG, or unique to MAGs with the same taxonomic classification on genus level were included in the analysis.

### Stable Isotope Cluster Analysis

Peptide identifications from ^12^C samples were used to identify isotopologue patterns of the respective peptides in mass spectra of corresponding ^13^C-labeled samples by comparing peptide masses, chromatographic retention times and MS/MS fragmentation patterns as previously described [23, 39]. Only mass spectral signals with a mass deviation below 10 p.p.m. and a retention time deviation below 3 min were selected. Isotopologue patterns were extracted manually from mass spectral data using the Xcalibur Qual Browser (v3.0.63, Thermo Fisher Scientific, Waltham, MA, USA). Isotopologue patterns were manually verified by comparisons between replicates and exclusion of patterns with signals before the monoisotopic peak (M-1 Da) or overlapping signals of other peptides. A total of 827 isotopologue patterns have been included in the analysis (Dataset S2).

The conventional approach of calculating the most probable ^13^C relative isotope abundance (RIA) of a peptide does not take into account the information contained in ^13^C isotopologue patterns, which allow a differentiation between direct utilization of a labeled carbon source and cross-feeding, as previously described [39]. To include this information in the analysis, we developed SIsCA. SIsCA was performed using R [31], with scripts being available on GitHub [40]. Measured isotopologue patterns for each peptide were compared to 21 predicted isotopologue patterns varying in ^13^C RIA (5% intervals from 0 to 100% ^13^C RIA), using an excel-based tool [40]. For each comparison between measured and predicted isotopologue pattern, a coefficient of determination (*R*^2^) was calculated as previously described [41]. In the resulting dataset of 21 *R*^2^ values per peptide (see Dataset S2), information from the original isotopologue pattern is retained, whereas at the same time, data from different peptides is comparable, time series can be integrated, and the dataset can easily be used for downstream statistical analysis. To differentiate microbes with different ^13^C isotopologue patterns, and hence different carbon sources, *R*^2^ values were averaged from samples obtained from replicate microcosms and peptides assigned to the same MAG. The resulting datasets of 21 *R*^2^ values per time point per MAG were visualized via PCA with the vegan software package [42]. PCA was used to reduce the dimensionality of the 21 *R*^2^ dataset, and within the resulting ordination space, MAGs with similar ^13^C isotopologue patterns (reflecting their carbon utilization profiles) clustered together. Clusters of MAGs with similar carbon utilization profiles were defined manually and validated by testing for overlapping confidence intervals.

Generation times of individual taxa were calculated by comparing the relative intensity of unlabeled and labeled peptide signals in mass spectrometric data, as previously described [23]. The number of doublings, *n*, was calculated according to Eq. [1] where $I_{12_{{{{{\mathrm{C}}}}}}}$ and $I_{13_{{{{{\mathrm{C}}}}}}}$ are the signal intensities of the unlabeled peptide and labeled peptide, respectively:

1 $$n = \log _2\frac{{I_{12_{{{{{\mathrm{C}}}}}}} + I_{13_{{{{{\mathrm{C}}}}}}}}}{{I_{13_{{{{{\mathrm{C}}}}}}}}}$$

If the mass spectrometric signals of unlabeled and labeled peptides overlapped, the monoisotopic peak was used to determine the total abundance of unlabeled peptide based on the natural distribution of heavy isotopes, as previously described [24]. Generation time, $t_{{{{{\mathrm{d}}}}}}$, was calculated with Eq. [2], where $\Delta t$ is incubation time:

2 $$t_{{{{{\mathrm{d}}}}}} = \frac{{\Delta t}}{n}$$

## Results

### Sulfur oxidation by active groundwater microbes

**Fig. 1:**
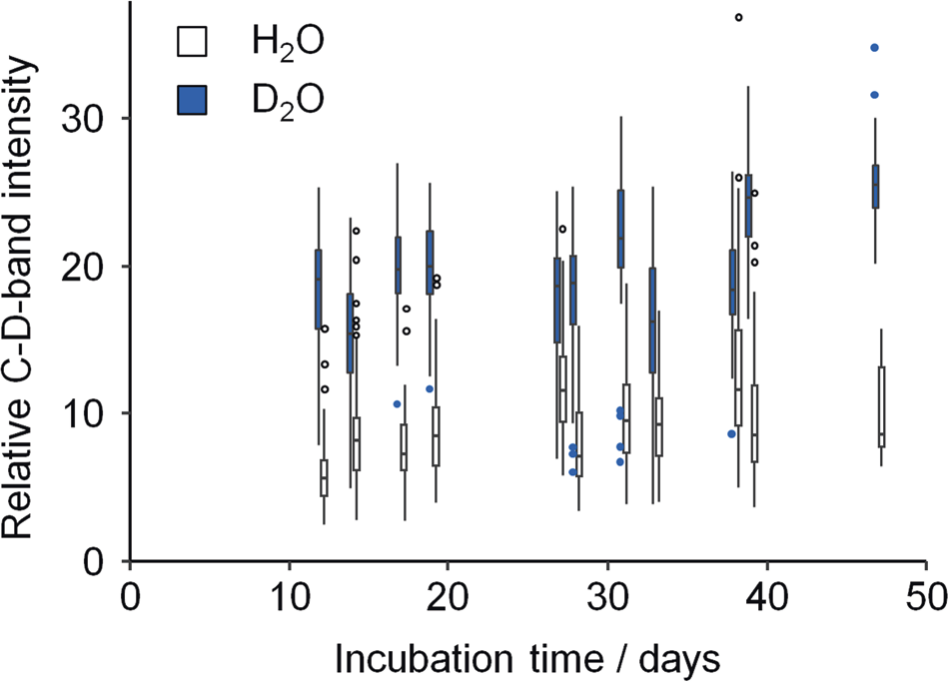
Quantification of deuterium incorporation by single-cell Raman microspectroscopy. Boxplots depict the relative intensity of Raman C-D bands, determined by A(C-D)/[A(C-D) + A(C-H)], from single-cell Raman spectra. Spectra were obtained from groundwater microcosms with 30% D_2_O (shaded) or H_2_O (empty) at various time points. Boxes show median, and first and third quartile; whiskers denote 5th and 95th percentile. Outliers are depicted as dots. A minimum of 147 spectra were obtained at each time point.

Groundwater microbiota responded immediately to the addition of thiosulfate, yielding increasing rates of sulfur oxidation. During the first three weeks of incubation, thiosulfate and oxygen consumption rates remained relatively low (1.7 ± 1.9 and 5.5 ± 2.0 µmol d^−1^ (mean ± SD), respectively; Fig. S1). Raman microspectroscopic analyses suggested that >95% of cells were active within the first 12 days of incubation. A distinct C-D band was observed at wavelength positions between 2100 and 2300 cm^−1^ in the single-cell Raman spectra of the groundwater microcosm amended with D_2_O (Fig. 1 and Fig. S2), which demonstrated new biomolecules were being synthesized by incorporating deuterium from D_2_O into C-D bonds. The relative intensity of the C-D band increased from 18.3% after 12 days to 25.7% after 47 days of incubation (median values; *p* < 2.2 × 10^−16^, *t* = −14.0, df = 142, two-sided Welch’s *t*-test), indicative of continued microbial proliferation and cross-feeding on deuterium-labeled organic carbon.

After 70 days of incubation, consumption rates of thiosulfate (7.2 ± 2.0 µmol d^−1^) and oxygen (12.8 ± 3.2 µmol d^−1^) had increased significantly (*p* = 6.48 × 10^−4^, *t* = 5.43, df = 7.90 [thiosulfate] and *p* = 1.27 × 10^−3^, *t* = 4.77, df = 8.30 [oxygen], two-sided Welch’s *t*-test, Fig. S1). Sulfate was produced at a consistent rate ranging between 8.1 and 9.6 µmol d^−1^ (no significant changes) throughout the duration of the experiment. Recorded stoichiometry for oxygen : thiosulfate : sulfate was roughly 2.8 : 1 : 2.6 over the course of incubation, very near the theoretical ratio of 2 : 1 : 2 for oxygen-dependent thiosulfate oxidation.

### Organism-specific ^13^C incorporation reveals distinct lifestyles

**Fig. 2:**
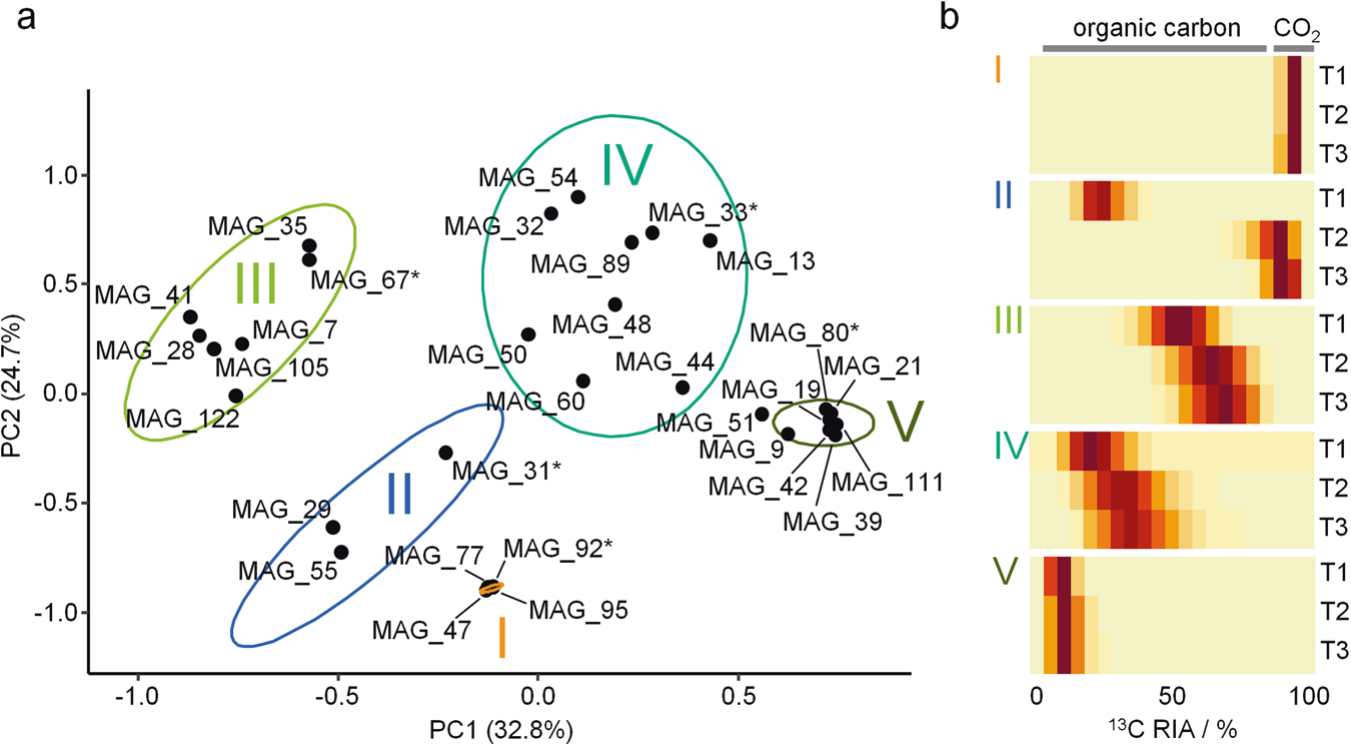
Clustering of selected MAGs based on carbon utilization. **a** Stable Isotope Cluster Analysis based on PCA of ^13^C incorporation profiles over incubation time obtained from SIP-metaproteomics of ^13^C-microcosm samples. Each point represents a distinct organism represented by one MAG. MAG clusters are indicated by Latin numbers. Ellipses depict 95% confidence intervals. All MAGs shown facilitated the acquisition of at least two replicates of ^13^C incorporation patterns per time point. **b** Representative ^13^C incorporation profiles of MAGs marked with asterisks are given for each cluster. Heatmaps depict the extent of ^13^C incorporation in peptides of the corresponding MAG after 21 (T1), 43 (T2), and 70 days (T3) of incubation (5% intervals, ranging from 0 to 100% ^13^C relative isotope abundance). Gray lines indicate the range of RIA expected for exclusive CO_2_ fixation and for assimilation of ^13^C-labeled organic carbon.

**Fig. 3:**
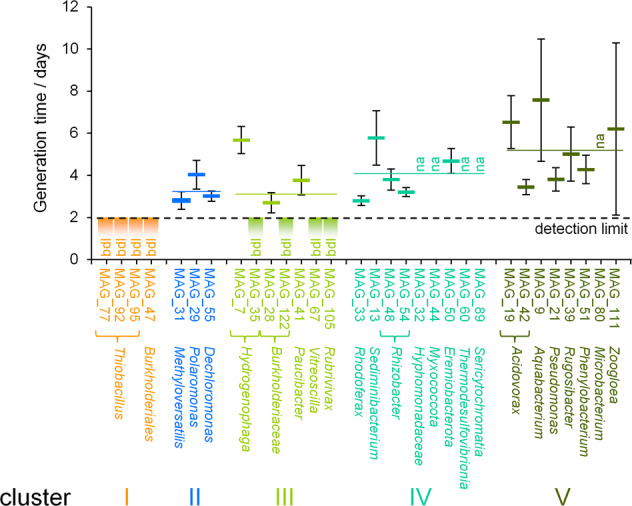
Generation times of groundwater microorganisms. Values were determined for the first 3 weeks of incubation, based on the relative abundance of ^12^C and ^13^C peptides. Shown are mean and SD based on *n* ≥ 4 replicate determinations. Colored horizontal lines indicate average generation time for each cluster. bdl: generation time fell below the detection limit of 2 days. na: quantification of generation time was not possible.

To address the carbon utilization schemes of key microbes, we conducted genome-resolved SIP-metaproteomic analyses after 21, 43, and 70 days of incubation. SIsCA then clustered the 31 most abundant MAGs into 5 distinct groupings, based on carbon utilization (Fig. 2, Fig. S3, and Dataset S2). Organisms represented by MAGs in cluster I were related to *Thiobacillus* (*Burkholderiales*) and exhibited a stable ^13^C RIA of 95% throughout the 70-day experiment (Fig. 2). We expected that microbes exclusively fixing CO_2_ would exhibit a high (>90%) and stable ^13^C RIA, as we replaced the majority of IC in the microcosms with ^13^C, and the release of unlabeled IC from mineralization should be negligible compared to the high labeled IC concentrations. The high ^13^C RIA observed hence indicated exclusive CO_2_ fixation. The deviation from 100% RIA is likely caused from incomplete replacement of IC with ^13^C. Although we cannot completely exclude the uptake of a very small amount of organic compounds, this is unlikely, as it would have resulted in a shift to higher RIA over incubation time, which we did not observe. The organisms from cluster I accounted for only 11% of the total number of MAGs across the five clusters, based on normalized coverage of the metagenomics dataset, and 3.2 ± 3.1% (mean ± SD) relative abundance in 16S rRNA gene copies (Fig. S4, Fig. S5, and Supplementary Information). By comparing the signal intensities of ^12^C- and ^13^C-enriched peptides, the generation time of these autotrophs was determined to be <2 days (Fig. 3), highlighting the rapid production of new ^13^C-labeled biomass from ^13^CO_2_.

Organisms represented by MAGs in cluster II were most closely related to species of *Methyloversatilis*, *Polaromonas*, and *Dechloromonas* (all *Burkholderiales*). These microbes exhibited a moderate 65% ^13^C RIA after 21 days of incubation (Fig. 2). However, after incubating for 43 and 70 days, ^13^C RIA increased to 91% (*p* = 1.57 × 10^−3^, *t* = −3.52, df = 26.5, two-sided Welch’s *t*-test; Fig. 2). The moderate RIA after 21 days indicated the utilization of unlabeled organic carbon from the groundwater. The ^13^C was likely derived from the uptake of labeled organic carbon produced by chemolithoautotrophs, but low rates of ^13^CO_2_ fixation cannot be ruled out either. The high RIA at 43 days indicated a switch to chemolithoautotrophic growth and no further uptake of organic carbon, comparable to cluster I. Exhibiting generation times between 2 and 4 days (Fig. 3), MAGs representing these mixotrophs were more than twice as abundant as those of cluster I, accounting for 26% of the total normalized coverage.

Over the first 21 days of incubation, mean ^13^C RIAs of cluster III and IV microbes increased from 65% to 76% and from 18% to 53%, respectively (*p* = 2.21 × 10^−13^, *t* = −8.50, df = 97.7 [cluster III] and *p* < 2.2 × 10^−16^, *t* = −11.6, df = 58.8 [cluster IV], two-sided Welch’s *t*-test; Fig. 2). This increasing trend of ^13^C RIA demonstrated two important aspects: first, it pointed to the assimilation of organic carbon, in part produced by chemolithoautotrophic metabolism of organisms of clusters I and II. Gradually shifting RIAs are a key indication of such cross-feeding in a SIP-metaproteomics experiment [22]. Nevertheless, in addition to the uptake of ^13^C organic carbon, the uptake of a small amount of ^13^CO_2_ cannot be excluded. Second, it hinted to an increased labeling of available organic carbon, through the fixation of ^13^CO_2_. Variations observed in ^13^C RIAs between species suggested different extents of cross-feeding on chemolithoautotrophically produced organic carbon, potentially due to preferences for different organic carbon compounds. Cluster III was the most abundant of all clusters, accounting for 28% of the total normalized coverage, whereas cluster IV accounted for 20% of this total. The vast majority of organisms represented by MAGs in these clusters exhibited generation times between 3 and 4 days (Fig. 3). However, cluster III microbes most closely related to species of *Hydrogenophaga*, *Vitreoscilla*, and *Rubrivivax* exhibited growth rates as fast as their cluster I counterparts.

In cluster V, average ^13^C RIAs reached 6% after 21 days of incubation and did not change thereafter, which hinted at active heterotrophic lifestyles early on in the experiment. Nonetheless, these organisms represented 15% of the total normalized coverage of all clusters. Generation times for cluster V microbes were slightly longer and more variable, ranging from 3.5 days for species of *Acidovorax* to 8 days for *Aquabacterium* spp. (Fig. 3).

Analyses of corresponding peptide RIAs of all analyzed MAGs showed that 43%, 68%, and 80% of the total microbial carbon was replaced with ^13^C following 21, 43, and 70 days of incubation, respectively. Quantitative DNA-SIP confirmed this labeling pattern via increases in the number of, and buoyant density shifts associated with, ^13^C-labeled operational taxonomic units (Fig. S6 and Supplementary Information). SIsCA suggested carbon transfer from autotrophic cluster I to mixotrophic cluster II, and from these two further to the organisms of cluster III through V through cross-feeding on ^13^CO_2_-derived organic carbon.

### Functional characterization of MAGs reveals putative mixotrophs

**Fig. 4:**
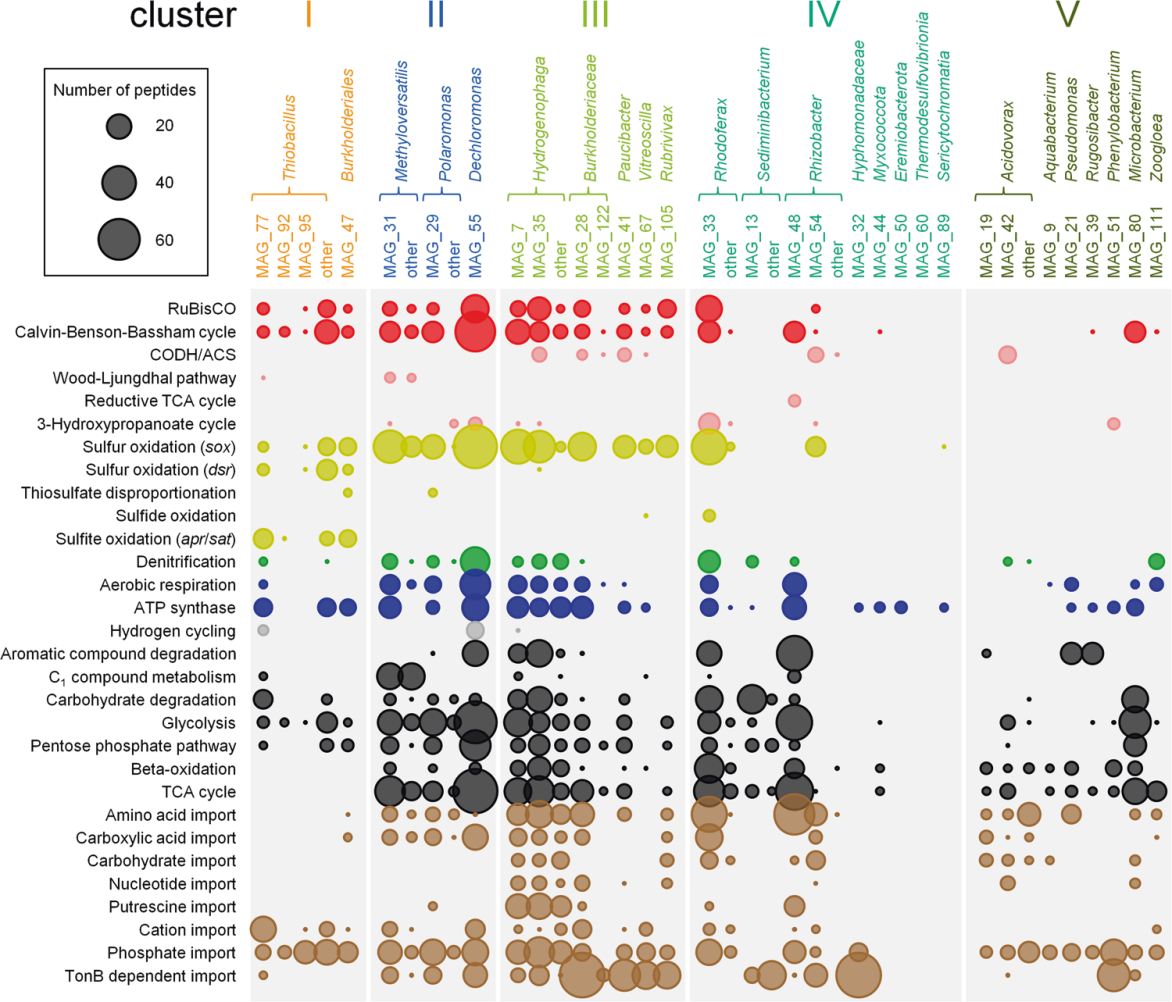
Metabolic functionality of selected MAGs. The sizes of the bubbles correspond to the total number of peptides detected for each MAG and each functional category identified at any time point (see Dataset S3). Metabolic functions are grouped into CO_2_ fixation (red), sulfur cycling (yellow), nitrogen cycling (green), aerobic respiration and ATP synthesis (blue), organic carbon utilization (black), and import functions (brown). The taxonomic categories “other” include peptides that were assigned to multiple MAGs affiliated with the same genus. Only MAGs considered in the Stable Isotope Cluster Analysis are shown. RuBisCO: ribulose-1,5-bisphosphate carboxylase/oxygenase, CODH/ACS: carbon monoxide dehydrogenase/acetyl-CoA synthase, TCA cycle: tricarboxylic acid cycle.

All of the putative autotrophs detected employed the Calvin–Benson–Bassham (CBB) cycle for CO_2_ fixation (Fig. 4). Subunits of the enzyme ribulose-1,5-bisphosphate carboxylase/oxygenase (RuBisCO) were detected on protein level for 15 of 31 MAGs, as well as additional enzymes of the CBB cycle for 14 of these. No other complete CO_2_ fixation pathways were identified. Proteins of the CBB cycle were present not only in organisms of cluster I (i.e., relatives of *Thiobacillus* spp.) or cluster II (e.g., relatives of *Methyloversatilis*, *Polaromonas*, and *Dechloromonas* spp.), but also in organisms most closely related to species of *Hydrogenophaga*, *Rhodoferax, Paucibacter*, and *Rubrivivax* of clusters III and IV. Although confirming the classification based on SIsCA of cluster I organisms as autotrophs and cluster II organisms as mixotrophs, this suggested that also organisms from cluster III and IV were able to assimilate ^13^CO_2_ alongside organic carbon, hence displaying a mixotrophic lifestyle. Mixotrophs thus comprised >50% of all microbial taxa represented across all clusters, which underscored the immense importance of their contributions to carbon cycling in the groundwater microcosms.

### MAGs express pathways for the utilization of reduced sulfur compounds

Sixteen MAGs expressed proteins for sulfur oxidation via the Sox or Dsr enzyme system (Fig. 4). Cluster II, III, and IV microbes phylogenetically affiliated with species of *Methyloversatilis*, *Dechloromonas*, *Hydrogenophaga*, *Rhodoferax*, and other *Betaproteobacteriales* utilized the Sox system exclusively. MAGs harbored gene clusters of the conserved *soxCDYZAXB* gene order (Fig. S7), featuring the core components of the Kelly-Friedrich pathway [43, 44]. This pathway facilitates the complete oxidation of thiosulfate to sulfate, without free intermediates [29]. Accessory genes *soxVW*, *soxEF*, *soxTRS*, and *soxH* were scattered through the MAGs disconnected from the main operon.

Cluster I microbes most closely related to *Thiobacillus* spp. produced enzymes for both the Sox and Dsr system, and corresponding MAGs housed a truncated *soxXYZAB* gene cluster that lacked genes *soxCD* required to oxidize the sulfane group of thiosulfate. As such, these organisms likely used the branched thiosulfate oxidation pathway typical for *Thiobacillus* spp. [45], whereby Dsr operating in reverse oxidizes the sulfane-derived sulfur atom to sulfite, with elemental sulfur as intermediate [29]. Cluster I MAGs maintained the conserved operon structure *dsrABEFHCMKLJOPNR*, including genes *dsrEFH* and *dsrL* typical for sulfur oxidizers but lacking gene *dsrD* for sulfate reduction [28]. These organisms also expressed *aprAB* and *sat*, which encode adenosine-5’-phosphosulfate reductase and ATP sulfurylase, respectively, each of which can function in reverse to oxidize sulfite to sulfate [46]. Hence, groundwater mixotrophs employed the Sox system to oxidize thiosulfate to sulfate, whereas chemolithoautotrophs from cluster I utilized an incomplete version of this system to oxidize the sulfone group and the Dsr/Apr/Sat system to oxidize the sulfane group of thiosulfate.

### Use of alternative electron acceptors and donors in sulfur oxidizers

Cytochrome c oxidase and other enzymes of the respiratory chain were detected in 15 sulfur oxidizer MAGs, 12 of which also harbored enzymes for nitrate reduction (i.e., nitrate reductase, nitrite reductase, nitric oxide reductase; Fig. 4). Several sulfur oxidizers related to species of *Dechloromonas* and *Rhodoferax* expressed both pathways concurrently. This suggested a widespread ability of the organisms in the groundwater microcosms to not only use oxygen, but also nitrate as electron acceptor. MAG_77 (*Thiobacillus*), MAG_55 (*Dechloromonas*), and MAG_7 (*Hydrogenophaga*) also expressed [NiFe]-hydrogenase genes.

### Utilization of organic carbon in oligotrophic groundwater

From cluster I to cluster III, the diversity of organic carbon compounds utilized by the MAGs increased. Whereas cluster I’s strict autotrophs only expressed pathways for sugar degradation, MAGs of clusters II through V produced proteins germane to the breakdown and transport of simple sugars (e.g., glycolysis, pentose phosphate pathway), amino acids (tricarboxylic acid cycle (TCA cycle)), fatty acids, C_1_ compounds, and aromatics (Fig. 4). The TCA cycle was one of the most abundant metabolic modules observed in MAGs of cluster II to V. Degradation pathways for toluene and ethylbenzene were expressed by organisms most closely related to species of *Dechloromonas* and *Rhizobacter*, respectively. Enzymes for naphthalene and catechol catabolism were detected in MAGs representing organisms related to *Hydrogenophaga* and *Pseudomonas* spp., whereas proteins for the degradation of complex carbohydrates (*e.g*., starch, chitin) were produced by MAGs representing relatives of *Microbacterium* and *Sediminibacterium* species. The metabolic machinery required to metabolize C_1_ compounds was detected primarily in microbes related to *Methyloversatilis* spp., which typically possessed methanol dehydrogenase, formate dehydrogenase, and other enzymes involved in tetrahydromethanopterin-dependent C_1_-cycling.

Gene products relevant to import systems for amino acids and carboxylic acids (e.g., alpha-keto acids, C_4_-dicarboxylates, lactate) were overly abundant in mixotrophs and heterotrophs of clusters II to V (Fig. 4). Cluster III to V microorganisms that had grown exclusively heterotrophically exhibited the greatest diversity of import-related proteins, including those for the transport of carbohydrates and nucleotides. Only transporters targeting cations (predominantly iron) and phosphate were detected in MAGs representing the autotrophs in cluster I.

## Discussion

**Fig. 5:**
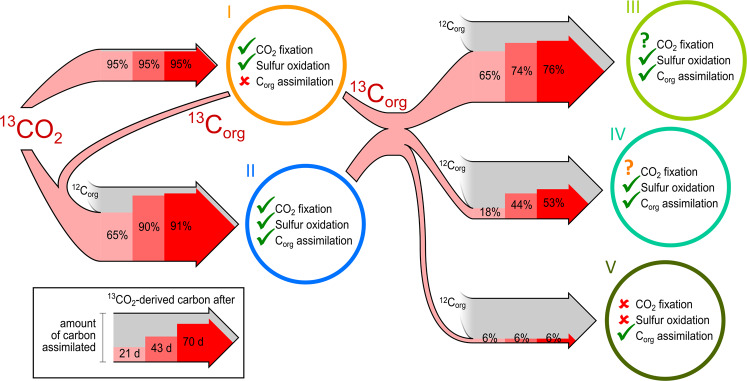
Carbon flux between microbial clusters. Red arrow inlays illustrate the fraction of ^13^CO_2_-derived carbon assimilated by each microbial cluster after 21, 43, and 70 days. Arrow width scales with the total amount of carbon assimilated based on the relative abundance of the respective microbial cluster in the metagenomics analysis. Fading gray arrows indicate uptake of unlabeled organic carbon from the groundwater. Checkmarks highlight the presence and activity of metabolic functions for CO_2_ fixation, utilization of organic carbon, and sulfur oxidation. The green question mark indicates potential activity, the orange question mark potential activity by only some members of the cluster.

Despite conditions strongly favoring autotrophic sulfur oxidizers, mixotrophs assimilating substantial amounts of organic carbon were the most abundant active microorganisms in the groundwater microcosms. With thiosulfate and oxygen readily available throughout the experiment, we expected a selective proliferation and predominance of chemolithoautotrophs exclusively assimilating CO_2_. Although a diverse microbial consortium was detected, such chemolithoautotrophs accounted for only 3% of this consortium (Fig. S4). Highly sensitive Raman spectroscopy showed that microbes were active at the outset of the incubation (no discernable lag phase), despite low sulfur oxidation rates. Genome-resolved SIP-metaproteomics combined with our novel SIsCA approach facilitated identification of active microbes, characterization of their expressed gene products (and linked pathways), and quantification of carbon uptake and transfer. From this, we inferred adaptations of groundwater organisms to oligotrophic conditions and their effect on fluxes of CO_2_-derived carbon within a diverse community over time (Fig. 5). Within 21 and 70 days of incubation, 43% and 80% of the total groundwater biomass consisted of CO_2_-derived carbon, respectively. Our results show that this rapid enrichment of CO_2_-derived carbon did not occur in fixed, linear progression from chemolithoautotrophs to heterotrophs, but through a highly complex and reticulated web of trophic interactions with mixotrophs as key players.

These mixotrophs were able to satisfy their carbon requirements by combining the fixation of CO_2_ with the uptake of organic carbon. Different microbial groups seemed to employ a variety of strategies to balance the input from these two carbon sources, which might increase their fitness when facing limited organic carbon in oligotrophic environments. Cluster II mixotrophs, e.g., transitioned from heterotrophy to autotrophy late in the incubation, potentially due to a limitation in organic carbon compounds accessible to them. In a similar vein, cluster III mixotrophs expressed pathways for autotrophic growth but assimilated organic carbon till the end of the incubation, likely because they were able to access a more diverse repertoire of carbon sources due to a greater metabolic versatility in organic carbon utilization. We postulate that these microbes used the CBB cycle for CO_2_ fixation to support heterotrophic growth by funneling excess reduction equivalents to CO_2_. This is known as a crucial support mechanism for heterotrophic growth in certain anaerobic phototrophic nonsulfur purple bacteria [47, 48]. In the groundwater, where terminal electron acceptors such as oxygen might become limiting at times, this strategy also could be useful to maintain redox balance and preserve carbon.

The ability for CO_2_ fixation allowed mixotrophs to grow considerably faster (generation times of 2 days or less) than cluster IV and V organisms restricted to an exclusively heterotrophic lifestyle (generation times up to 8 days). Unexpectedly, the heterotrophs in cluster IV and V were also able to oxidize reduced sulfur compounds, suggestive of a chemolithoheterotrophic lifestyle. In the Hainich CZE, but also in modern groundwaters beyond, weathering of interspersed pyrite minerals from the rocks of the aquifers leads to a constant release of reduced sulfur compounds [25–27]. This should render sulfur oxidation an attractive alternative for groundwater microbes to the oxidation of organic compounds for energy conservation.

The diversity of organic carbon utilization motifs shifted gradually, and inversely, with CO_2_ fixation. At one end of the transition were the strict autotrophs of cluster I, relying exclusively on CO_2_ as carbon source. No organic carbon transporters were detected on protein level for any of these organisms. Their limited metabolic breadth restricted growth to that from simple sugars, likely to utilize carbon assimilated via the CBB cycle [49]. At the other end of the transition were organisms from clusters IV and V, which assimilated organic carbon exclusively. To endure the oligotrophic environment *sans* autotrophic CO_2_ fixation machinery, these organisms had to maintain and express a wide variety of organic carbon transport and assimilation pathways. However, the most abundant organisms in the groundwater microcosms were the mixotrophs of clusters II and III. Exploiting their physiological flexibility enabled them to outnumber their autotrophic brethren in cluster I in a ratio of 5 to 1. The observed variations in ^13^C RIA between mixotrophic species might be caused by the use of different carbon compounds. Accessing different organic carbon pools might also explain why some organisms switched to CO_2_ fixation whereas others continued to use organic carbon. Differentiated substrate spectra would also allow organisms to avoid competition and exist in parallel niches, leading to increased fitness in oligotrophic groundwater.

In a recent metagenomics study at the Hainich CZE, ~12% of reads mapped to MAGs of putative chemolithoautotrophs in the groundwater of well H41, whereas ~27% mapped to MAGs of heterotrophs [50]. Organisms of the order *Burkholderiales*, related to the key mixotrophic taxa *Methyloversatilis*, *Polaromonas*, and *Dechloromonas* in our groundwater microcosms, gave rise to the greatest number of RuBisCO-encoding transcripts at this site, and RuBisCO was found in up to 17% of the community [17]. RuBisCO genes were also detected in 85% of groundwater samples obtained from multiple locations in Germany and Austria [51]. In agreement with this wealth of genetic information, rates of CO_2_ fixation under in situ conditions in the groundwater were close to those reported from the photic zone of oligotrophic marine waters [50]. The recent finding that the metabolic potential for CO_2_ fixation coincides with the complexity of subsurface food webs in the Hainich CZE [52] suggests that this essential function has consequences for the entire ecosystem. We speculate that the presence of the CBB cycle might not only facilitate the fixation of CO_2_, but also the utilization of organic carbon.

Sulfur oxidizers formed the largest part of the chemolithoautotrophic community in the Hainich CZE under oxygen limited conditions, whereas under oxic condition, nitrifying chemolithoautotrophs were more abundant [50]. The higher abundance of sulfur oxidizers under oxygen limited conditions might be linked to the benefits of using the CBB cycle to maintain redox balance. Certain sulfur oxidizers, such as *Rhodoferax* and *Sediminibacterium*, but also other genera such as *Sulfuritalea* and *Acidiferrobacteraceae*, are core species present in each groundwater well [53]. The wider range of diversity of sulfur oxidizers in situ compared to our groundwater microcosms may suggest additional strategies for balancing organic carbon and CO_2_ fixation that allow organisms to coexist, with resulting niches not realized in the microcosms.

For taxa such as *Polaromonas*, *Dechloromonas*, *Hydrogenophaga*, and *Rhodoferax* spp., the ability to oxidize sulfur has been posited based solely on genomic evidence [54–57]. Hitherto, chemolithoautotrophic growth on reduced sulfur compounds has not been observed from any of these genera in pure culture. Our study demonstrates that these organisms can use reduced sulfur as an energy source, and species of *Polaromonas*, *Dechloromonas*, and potentially *Hydrogenophaga* used it to fuel autotrophic growth. These sulfur oxidizers expressed pathways for both aerobic respiration and denitrification, despite the fact that no nitrate was added and nitrate concentrations in the groundwater of this well never exceeded 10 mg/L [27]. Constitutive maintenance and expression of denitrification enzymes is likely more energetically cost effective than regulating gene expression [58]. This strategy also affords these microbes the advantage of utilizing different electron acceptors when oxygen becomes limited.

*Thiobacillus* spp. related to the organisms of cluster I are described to grow strictly autotrophic as they utilize an incomplete TCA cycle that precludes heterotrophic growth [59]. *Thiobacillus* can store the elemental sulfur produced as intermediate by the Dsr enzyme system in periplasmic granules [59, 60]. This storage might allow the organism to withstand times where no reduced sulfur compounds in the groundwater are available. Previously, by carrying out thiosulfate- and hydrogen-driven denitrification, *Thiobacillus* spp. grew up to represent upwards of 50% of an enrichment culture obtained from Hainich CZE groundwater [30]. In situ, however, *Thiobacillus* spp. are typically found in lower numbers [17] and most commonly appear in deeper, more CO_2_-rich subsurface systems [13]. This suggests that its ecological niche provides it with fewer opportunities in oligotrophic modern groundwater compared to the more physiologically versatile mixotrophs.

There are two key advantages to being a mixotrophic sulfur oxidizer in the groundwater habitat. First, these cells exist completely independent of surface carbon input dynamics. The energy sources they rely on are released autochthonously into the groundwater from pyrite minerals in the geological setting of the aquifer. Second, their diverse breadth of physiological capabilities allows these microbes to modulate the means by which they satisfy their anabolic requirements and energy demands based on the types of carbon available. This includes carbohydrate degradation pathways for surface-derived plant polymers [8, 61], amino acid, and nucleotide uptake systems for microbially derived carbon [62, 63], C_1_ metabolic functions for C_1_ carbon compounds from biomass degradation [64], and hydrocarbon degradation pathways for rock-derived carbon [65, 66]. The mixotrophic lifestyle, by no means a rare or insignificant trait in the groundwater, thus appears to bestow fitness on the microbes. We hypothesize that similar strategies exploiting a myriad of carbon assimilation and versatile energy acquisition pathways benefit microbes in other oligotrophic systems, such as boreal lakes or the upper ocean [67, 68].

## Conclusions

Our novel SIsCA-based approach facilitated the quantitative and temporal resolution of carbon flux through microbial key populations in microcosms with modern groundwater. Mixotrophs were the most abundant group, employing a range of strategies to balance CO_2_ fixation and organic carbon uptake that potentially provided fitness under oligotrophic conditions. This CO_2_-derived organic carbon was rapidly incorporated into, and recycled throughout, microbial biomass through a highly efficient and complex trophic network. To mitigate low levels of organic carbon, autotrophic, mixotrophic, and heterotrophic microorganisms utilized reduced sulfur compounds as energy sources and preserved what organic carbon was available for anabolic demands. A wide variety of carbon assimilation pathways enabled mixotrophs and heterotrophs to make optimal use of the scarce amounts of organic carbon characteristic of modern groundwater in our microcosms. We posit that the concerted deployment of a wide variety of highly versatile pathways for assimilating carbon and generating energy from inorganic sources is key to microbial success in oligotrophic environments. The findings of this investigation significantly enhance our understanding of microbial survival strategies and their role in ecosystem functioning while demonstrating the powerful utility of next-generation physiology approaches such as SIsCA in testing hypotheses established in metagenomics-based endeavors.

## Supplementary information

Supplementary Information

Dataset S1

Dataset S2

Dataset S3

## Data Availability

Metagenomic and amplicon sequencing data are available at NCBI under BioProject accession PRJNA633367. Mass spectrometry proteomics data have been deposited into the ProteomeXchange Consortium via the PRIDE [69] partner repository with the dataset identifier PXD024889. For review, these data can be accessed with username “reviewer_pxd024889@ebi.ac.uk” and password “Bnvcsmie”.
