# Supplementary material for: Bolstering fitness via CO2 fixation and organic carbon uptake: mixotrophs in modern groundwater
Source: ISME J. 2021 Dec 7;16(4):1153–62. doi: 10.1038/s41396-021-01163-x (PMC8941145; doi:10.1038/s41396-021-01163-x)
Supplement: Supplementary file 1 — Supplementary Information [file 41396_2021_1163_MOESM1_ESM.pdf]

Supplementary Methods

Conditions at the groundwater sampling site

Well H41 of the Hainich Critical Zone Exploratory (CZE) provides access to an aquifer assemblage at 48 m depth in a trochite limestone stratum. Sourced by a beech forest (*Fagus sylvatica*) recharge area, the oxic groundwater in this well maintains mean dissolved oxygen concentrations of 5.0 ± 1.5 mg L^{-1}, < 0.1 mg L^{-1} ammonium, 1.9 ± 1.5 mg L^{-1} dissolved organic carbon, 70.8 ± 12.7 mg L^{-1} total inorganic carbon, and a pH of 7.2 (1, 2).

Hydrochemical analyses

While incubating the 18 microcosms supplemented with ^{12}C- or ^{13}C-bicarbonate, concentrations of oxygen, thiosulfate, and sulfate were determined at regular intervals. Oxygen concentrations were determined using a contactless fiber-optic oxygen sensor (Fibox 4 trace with SP-PSt3-SA23-D5-YOP-US dots [PreSens Precision Sensing GmbH, Regensburg, Germany]). Measurements were collected from three ^{12}C microcosms and three ^{13}C microcosms every two days for the first three weeks, and once weekly thereafter. Thiosulfate concentrations were determined via colorimetric titration assays with iodine (3). Samples from all microcosms were evaluated every four to seven days. For each measurement, 2 mg potassium iodide was mixed into 1 mL of sample, followed by the addition of 10 µL of zinc iodide-starch solution (4 g L^{-1} starch, 20 g L^{-1} zinc chloride and 2 g L^{-1} zinc iodide) and 10 µL of 17% (v:v) phosphoric acid. Titration was performed by adding 5 µL of 0.005 N iodine at a time until the solution turned faint blue. Thiosulfate concentrations ($c_{thiosulfate}$ in mg L^{-1}) were then calculated according to equation (I), where V_{iodine} is the volume of iodine solution added and V_{sample} is the sample volume:

$$c_{thiosulfate} = \frac{V_{iodine} \times 561}{V_{sample}} \quad \text{(I)}$$

Sulfate concentrations were determined via a turbidimetric assay (4) from all microcosms every four to seven days. For each measurement, 1 mL of either microcosm sample, standard (50 µM to 1000 µM potassium sulfate) or blank (dH_2O) was mixed with 0.4 mL 0.5 M HCl and 0.2 mL $BaCl_2$-gelatin reagent (0.5 g gelatin and 8 g $BaCl_2$ in 200 mL dH_2O). Following 1 h incubation in the dark, absorbances were measured at 420 nm in a DR3900 spectrophotometer (HACH, Düsseldorf, Germany).

Amplicon sequencing

For taxonomic characterization of the bacterial community in the microcosms, amplicon sequencing of the bacterial 16S rRNA gene, region V3 to V5, was done. Polymerase chain reaction was performed using primer pair Bact_341F/Bact_805R (5) and HotStarTaq Mastermix (Qiagen, Hilden, Germany) as described previously (6). Amplicons were purified using NucleoSpin Gel & PCR Clean-Up Kit (Macherey-Nagel, Düren, Germany). The NEBNext Ultra DNA Library Prep Kit for Illumina (New England Biolabs, Frankfurt, Germany) was used to prepare libraries for amplicon sequencing, following the manufacturer’s instructions. Amplicons were purified using AMPure XP Beads (Beckman Coulter, Krefeld, Germany), and amplicon sequencing was then carried out in-house on a MiSeq platform (Illumina, Eindhoven, The Netherlands) with v3 chemistry.

Raw sequence data was analyzed using mothur (v.1.39) (7), according to the mothur standard operating procedures (8) as previously described (9). OTU binning with a 3% identity cutoff was performed, followed by OTU classification using the SILVA reference database release SSU 132 (10). Raw MiSeq sequencing data have been deposited in the Sequence Read Archive (SRA) of NCBI under BioProject accession PRJNA633367.

Assessment of DNA density shifts

To evaluate the ^{13}C incorporation on nucleic acid level, DNA samples extracted from the triplicate groundwater microcosms supplemented with ^{13}C and ^{12}C bicarbonate after 21 and 70 days of incubation were subjected to SIP ultracentrifugation in CsCl gradients as previously described (11).

Ultracentrifugation was carried out in an NVT 90 rotor (Beckman Coulter, Brea, CA, USA) in a Sorvall Discovery 90SE ultracentrifuge (Thermo Fisher Scientific, Waltham, MA, USA) at 40,900 rpm and 20 °C for 60 h. The gradients were separated into 12 to 14 fractions, covering a buoyant density range from 1.77 g ml^{-1} to 1.68 g ml^{-1}. The buoyant density of each fraction was determined using a Reichert AR200 digital refractometer (Reichert Analytical Instruments, Depew, NY, USA). To account for tube-to-tube and spin-to-spin variations of the density gradient (12), gradients were normalized by centering the density of ^{12}C DNA onto 1.70 g ml^{-1}. The DNA in the density fractions was purified by precipitation with NaCl-PEG as previously described (13) and quantified fluorometrically using the Qubit dsDNA broad-range assays (Thermo Fisher Scientific). Subsequently, amplicon sequencing of the bacterial 16S rRNA gene was performed as described above. OTU-wise DNA buoyant density profiles over the gradients were obtained as previously described (14). Only OTUs that were represented by at least 10 reads in one fraction of each ^{12}C and each ^{13}C replicate were included in the analysis. This was done separately for both time points. From the DNA buoyant density profiles of each OTU, the DNA density of that OTU ($\overline{\rho}_{OTU}$) was determined by performing least-squares regression of the OTU abundance A_{OTU} per fraction f to the fraction density ρ_f using a nonlinear model (II), where α^2 represents the variance of the OTU DNA density. An R script for calculation is available on GitHub (15).

$$A_{OTU,f} = e^{-\frac{\left(\rho_f - \overline{\rho}_{OTU}\right)^2}{2\alpha^2}} \qquad \text{(II)}$$

The average ^{12}C and ^{13}C OTU DNA density was determined as the mean of the respective triplicates. The density shift between ^{12}C and ^{13}C samples was determined separately for each time point. The significance of the shift was assessed using Student's *t*-test based on the respective triplicates.

Generation of MAGs from sequencing data

Raw sequencing data was quality filtered using BBDuk (16) and subjected to assembly with metaSPAdes v3.13.0 (17). Applying only contigs greater than 1,000 bp in length, three different algorithms facilitated genomic binning: MaxBin 2.0 v2.2.7 (18), MetaBAT 2 v2.12.1 (19), and

BinSanity v0.2.7 (20). Bin refinement was accomplished using the MetaWRAP pipeline v1.1.3 (21). Only bins that were both more than 50% complete and contained less than 10% contamination were considered. Bins were classified with GTDB-Tk v0.3.2 (22), and completeness parameters were appraised with CheckM v1.0.12 (23). Bins from different samples were dereplicated using FastANI v1.0 (24). The Prokka pipeline v1.13.3 (25) using the --metagenome option and the Prokka database (2019_09) based on SwissProt was used to assign functional annotations to gene sequences and to translate into amino acid sequences for metaproteomics analysis. Metagenomic bins of particular interest (per metaproteomics analysis) were manually refined with Anvi'o v6.1 (26), rendering the completed MAGs. Normalized coverage values for all MAGs were calculated by dividing raw coverage values by the relative abundance of *rpoB* genes in each metagenome. Gene abundances of *rpoB* were determined using ROCker (27).

Supplementary Results

Functional composition of the whole microbial community

Mapping the functional information obtained by SIsCA and genome-resolved metaproteomics to the corresponding taxa in 16S rRNA gene profiles allowed us to classify the lifestyle of up to 50% of the microbial community (Figure S4). Over all time points, only 3.2 ± 3.1% (mean ± sd) of the community were composed of strict autotrophs, primarily affiliated with *Thiobacillus*. Mixotrophs comprised 17.6 ± 4.3% of the total community and were dominated by *Rhodoferax* and *Hydrogenophaga*, but the largest fraction of the total community, with 20.1 ± 7.8%, consisted of heterotrophs, primarily affiliated to *Sediminibacterium* (*Bacteroidetes*), *Pseudomonas* (*Gammaproteobacteria*), *Sericytochromatia* (*Cyanobacteria*) and *Microbacterium* (*Actinobacteria*) (Figure S5).

DNA-SIP supports role of $^{13}CO_2$-derived carbon

In addition to the Stable Isotope Cluster Analysis (SIsCA) approach based on metaproteomics data, quantitative DNA-SIP was performed to provide a higher coverage of the microbial community. The

number of ^{13}C-labeled taxa increased from 21 OTUs after 21 days of incubation to 65 OTUs after 70 days of incubation, observable by a significant shift of DNA buoyant density (Figure S6). While after 21 days, these OTUs were mainly affiliated with *Burkholderiales* such as *Thiobacillus*, *Hydrogenophaga* and *Polaromonas*, after 70 days, various other *Alpha*- and *Gammaproteobacteria* were included. The average buoyant density shift of these OTUs likewise increased significantly from 0.021 ± 0.010 g ml^{-1} to 0.028 ± 0.013 g ml^{-1} (p = 0.014, t = -2.28, df = 38.3, one-sided Welch's t-test) in this period. This highlights the increasing role of $^{13}CO_2$-derived carbon introduced by chemolithoautotrophic activity into the microbial carbon pool in the groundwater incubations, and the flux of ^{13}C through the microbial food web.

References

1. Kohlhepp B, Lehmann R, Seeber P, Küsel K, Trumbore SE, Totsche KU. Aquifer configuration and geostructural links control the groundwater quality in thin-bedded carbonate-siliciclastic alternations of the Hainich CZE, central Germany. *Hydrol Earth Syst Sc* 2017; **21**(12): 6091-116.

2. Schwab VF, Herrmann M, Roth VN, Gleixner G, Lehmann R, Pohnert G *et al.* Functional diversity of microbial communities in pristine aquifers inferred by PLFA- and sequencing-based approaches. *Biogeosciences* 2017; **14**(10): 2697-714.

3. DEV-D15. Deutsche Einheitsverfahren zur Wasser-, Abwasser- und Schlammuntersuchung. Physikalische, chemische, biologische und bakteriologische Verfahren. Weinheim, Germany: VCH Verlagsgesellschaft; 1975.

4. Tabatabai M. A rapid method for determination of sulfate in water samples. *Environ Lett* 1974; **7**(3): 237-43.

5. Herlemann DPR, Labrenz M, Jürgens K, Bertilsson S, Waniek JJ, Andersson AF. Transitions in bacterial communities along the 2000 km salinity gradient of the Baltic Sea. *ISME J* 2011; **5**(10): 1571-9.

6. Kumar S, Herrmann M, Blohm A, Hilke I, Frosch T, Trumbore SE *et al.* Thiosulfate- and hydrogen-driven autotrophic denitrification by a microbial consortium enriched from groundwater of an oligotrophic limestone aquifer. *FEMS Microbiol Ecol* 2018; **94**(10): fiy141.

7. Schloss PD, Westcott SL, Ryabin T, Hall JR, Hartmann M, Hollister EB *et al.* Introducing mothur: Open-source, platform-independent, community-supported software for describing and comparing microbial communities. *Appl Environ Microb* 2009; **75**(23): 7537-41.

8. Kozich JJ, Westcott SL, Baxter NT, Highlander SK, Schloss PD. Development of a dual-index sequencing strategy and curation pipeline for analyzing amplicon sequence data on the MiSeq Illumina sequencing platform. *Appl Environ Microb* 2013; **79**(17): 5112-20.

9. Taubert M, Stöckel S, Geesink P, Girnus S, Jehmlich N, von Bergen M *et al.* Tracking active groundwater microbes with D_2O labelling to understand their ecosystem function. *Environ Microbiol* 2018; **20**(1): 369-84.

10. Quast C, Pruesse E, Yilmaz P, Gerken J, Schweer T, Yarza P *et al.* The SILVA ribosomal RNA gene database project: improved data processing and web-based tools. *Nucleic Acids Res* 2013; **41**(D1): D590-D6.

11. Neufeld JD, Vohra J, Dumont MG, Lueders T, Manefield M, Friedrich MW *et al.* DNA stable-isotope probing. *Nat Protoc* 2007; **2**(4): 860-6.

12. Sieradzki ET, Koch BJ, Greenlon A, Sachdeva R, Malmstrom RR, Mau RL *et al.* Measurement error and resolution in quantitative stable isotope probing: implications for experimental design. *bioRxiv* 2020; e-pub ahead of print Epub; doi:10.1101/2020.02.25.965764.

13. Taubert M, Stähly J, Kolb S, Küsel K. Divergent microbial communities in groundwater and overlying soils exhibit functional redundancy for plant-polysaccharide degradation. *Plos One* 2019; **14**(3).

14. Taubert M, Grob C, Howat AM, Burns OJ, Pratscher J, Jehmlich N *et al.* Methylamine as a nitrogen source for microorganisms from a coastal marine environment. *Environ Microbiol* 2017; **19**(6): 2246-57.

15. Taubert M. qSIP. 2020 [updated 23.10.2020; cited 2021]; Available from: https://github.com/m-taubert/qSIP.

16. Bushnell B. BBTools software package. 2014 [updated 03.02.2021; cited 2020]; Available from: http://sourceforge.net/projects/bbmap.

17. Nurk S, Meleshko D, Korobeynikov A, Pevzner PA. metaSPAdes: a new versatile metagenomic assembler. *Genome Res* 2017; **27**(5): 824-34.

18. Wu YW, Simmons BA, Singer SW. MaxBin 2.0: an automated binning algorithm to recover genomes from multiple metagenomic datasets. *Bioinformatics* 2016; **32**(4): 605-7.

19. Kang DWD, Li F, Kirton E, Thomas A, Egan R, An H *et al.* MetaBAT 2: an adaptive binning algorithm for robust and efficient genome reconstruction from metagenome assemblies. *Peerj* 2019; **7**(e7359): 1-13.

20. Graham ED, Heidelberg JF, Tully BJ. BinSanity: unsupervised clustering of environmental microbial assemblies using coverage and affinity propagation. *Peerj* 2017; **5**(e3035): 1-19.

21. Uritskiy GV, DiRuggiero J, Taylor J. MetaWRAP - a flexible pipeline for genome-resolved metagenomic data analysis. *Microbiome* 2018; **6**: 1-13.

22. Chaumeil PA, Mussig AJ, Hugenholtz P, Parks DH. GTDB-Tk: a toolkit to classify genomes with the Genome Taxonomy Database. *Bioinformatics* 2019; **36**(6): 1925-7.

23. Parks DH, Imelfort M, Skennerton CT, Hugenholtz P, Tyson GW. CheckM: assessing the quality of microbial genomes recovered from isolates, single cells, and metagenomes. *Genome Res* 2015; **25**(7): 1043-55.

24. Jain C, Rodriguez RL, Phillippy AM, Konstantinidis KT, Aluru S. High throughput ANI analysis of 90K prokaryotic genomes reveals clear species boundaries. *Nat Commun* 2018; **9**(1): 1-8.

25. Seemann T. Prokka: rapid prokaryotic genome annotation. *Bioinformatics* 2014; **30**(14): 2068-9.

26. Eren AM, Esen OC, Quince C, Vineis JH, Morrison HG, Sogin ML *et al.* Anvi'o: an advanced analysis and visualization platform for 'omics data. *Peerj* 2015; **3**(e1319): 1-29.

27. Orellana LH, Rodriguez-R LM, Konstantinidis KT. ROCker: accurate detection and quantification of target genes in short-read metagenomic data sets by modeling sliding-window bitscores. *Nucleic Acids Res* 2017; **45**(3): e14.

Supplementary Figure and Table legends

Figure S1: Hydrochemical conditions in groundwater microcosms. (A) Mean values for oxygen, thiosulfate and sulfate concentrations determined in all ^{12}C and ^{13}C microcosms over incubation time are given. Linear regression curves are shown for 0 to 21 days (dashed line), 21 to 43 days (dotted line) and 43 to 70 days (solid line). (B) Rates of oxygen and thiosulfate consumption as well as sulfate production based on linear regression are summarized over three time intervals of incubation. The number of replicates is n=18 for 0 to 21 days, n=12 to 43 days and n=6 to 70 days. Error bars indicate standard deviation.

Figure S2: Raman microspectroscopic analysis of the groundwater microbial community. (A) Mean Raman spectra of groundwater bacteria incubated with heavy water (D_2O) for 12 to 47 days. (B) Mean Raman spectra of groundwater bacteria incubated with H_2O for 12 to 47 days. (C) Confusion matrix showing the results of the PCA-LDA model built for the classification of metabolically active bacterial cells in groundwater. The differentiation between deuterium labeled (D_2O) and non-labeled (H_2O) bacterial cells achieved an overall accuracy of 92.2%, a mean sensitivity of 92.6% and a mean specificity of 92.6%.

Figure S3: Stable Isotope Cluster Analysis (SIsCA) of peptides assigned to MAGs. The analysis is based on PCA of ^{13}C incorporation profiles over incubation time obtained by SIP-metaproteomics of samples from the ^{13}C-microcosms. Each point represents one peptide of an organism associated with a particular MAG. The color code is used to highlight peptides that belong to the same MAG.

Figure S4: Functional composition of the groundwater microbial community. Shown are relative abundances of autotrophs (cyan), mixotrophs (blue), and heterotrophs (orange) of the groundwater microcosms on DNA level. The relative quantification of microbial taxa is based on bacterial 16S rRNA gene amplicon sequencing data. Functional classification of microbial taxa into the functional groups is based on the results of the SIP-metaproteomics analysis.

Figure S5: Taxonomic composition of the microbial community in the groundwater microcosms. Shown are the 20 most abundant bacterial genera based on read abundance from MiSeq amplicon sequencing of bacterial 16S rRNA genes. Further genera are summarized in the category 'others'. Each bar corresponds to one replicate microcosm. One replicate from the microcosms incubated for 21 days is not shown as the sequencing of the respective sample failed.

Figure S6: Change of DNA buoyant density for bacterial OTUs in the groundwater microcosms. Density shifts are indicative of ^{13}C incorporation in DNA of the respective organism represented by the OTU after 21 days (grey) or 70 days (red) of incubation. Shifts were calculated as difference between density in triplicate ^{12}C samples and density in triplicate ^{13}C samples. Error bars represent standard deviation of the density shift. Only OTUs with significant density shifts are shown. OTUs are sorted by decreasing density shift individually for each time point. The solid lines indicate the average density shift of the shown OTUs, the dashed lines indicate the respective standard deviation.

Figure S7: Gene clusters involved in sulfur oxidation observed in MAGs obtained from the groundwater microcosms. (A) Clusters of *sox* genes observed in MAGs related to *Thiobacillus* showing a canonical *soxXYZAB* gene order. The *sox* cluster of *Thiobacillus denitrificans* ATCC 25259 (NC_007404.1) is given as reference. (B) Clusters of *sox* genes observed in other MAGs showing a canonical *soxCDYZAXB* gene order. The *sox* cluster of *Dechloromonas aromatica* RCB (CP000089.1) is given as reference. (C) Clusters of *dsr* genes observed in the MAGs. The *dsr* gene cluster of *Thiobacillus denitrificans* ATCC 25259 is given as reference. Genes are represented by arrows. Grey arrows show unspecific genes. For arrows with a red outline, products of the corresponding genes have been detected by metaproteomics analysis. Numbers above the arrows indicate the respective contigs of the MAGs or the gene accession numbers for references. Lines between arrows indicate gaps larger than 100 nucleotides. Ellipses ('…') indicate splits between contigs or gaps of more than 5,000 nucleotides on one contig. Scale bar depicts gene length of 1,000 nucleotides. Time point and replicate of the sample the respective MAG was obtained from is shown below the MAG description.

Table S1: Accession numbers and taxonomic affiliation of metagenome-assembled genomes (MAGs).

Figure S1:

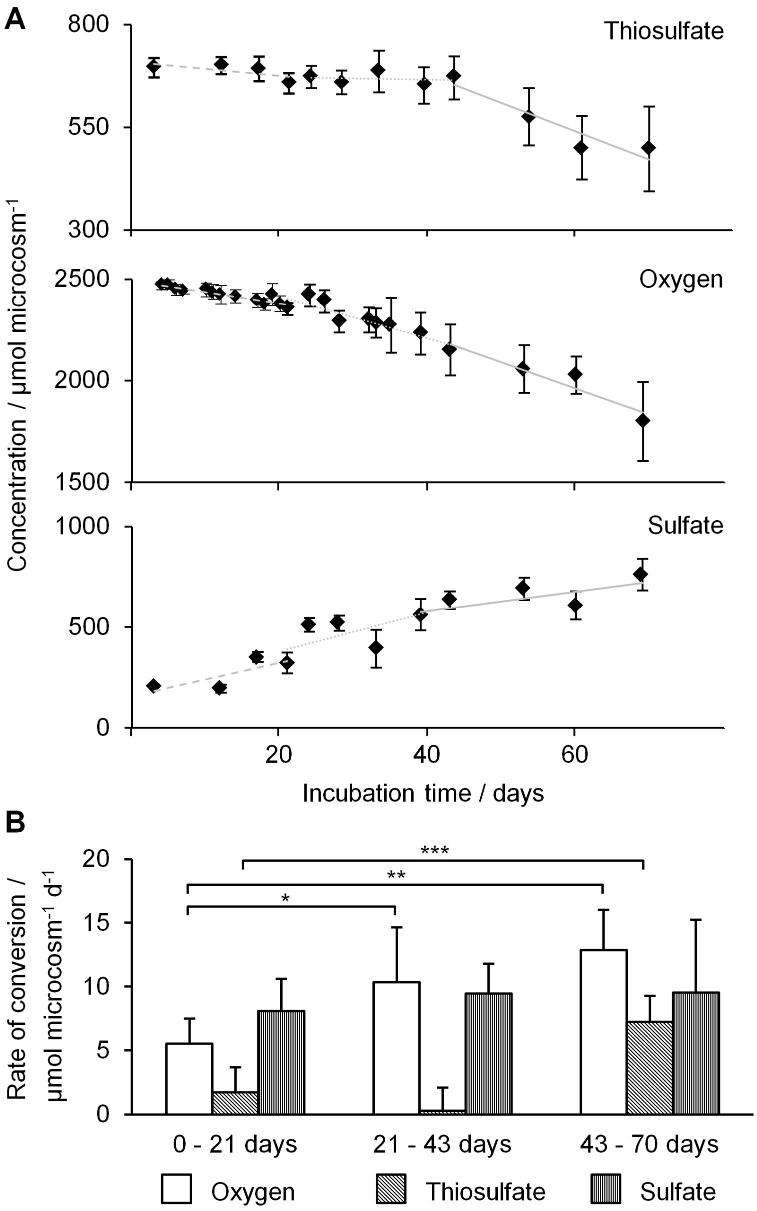
A
Thiosulfate
Oxygen
Sulfate
800
550
300
2500
2000
1500
1000
500
0
20
40
60
Concentration / µmol microcosm-1
Incubation time / days
B
Rate of conversion / µmol microcosm-1 d-1
20
15
10
5
0
***
**
*
0 - 21 days
21 - 43 days
43 - 70 days
Oxygen
Thiosulfate
Sulfate

Figure S2:

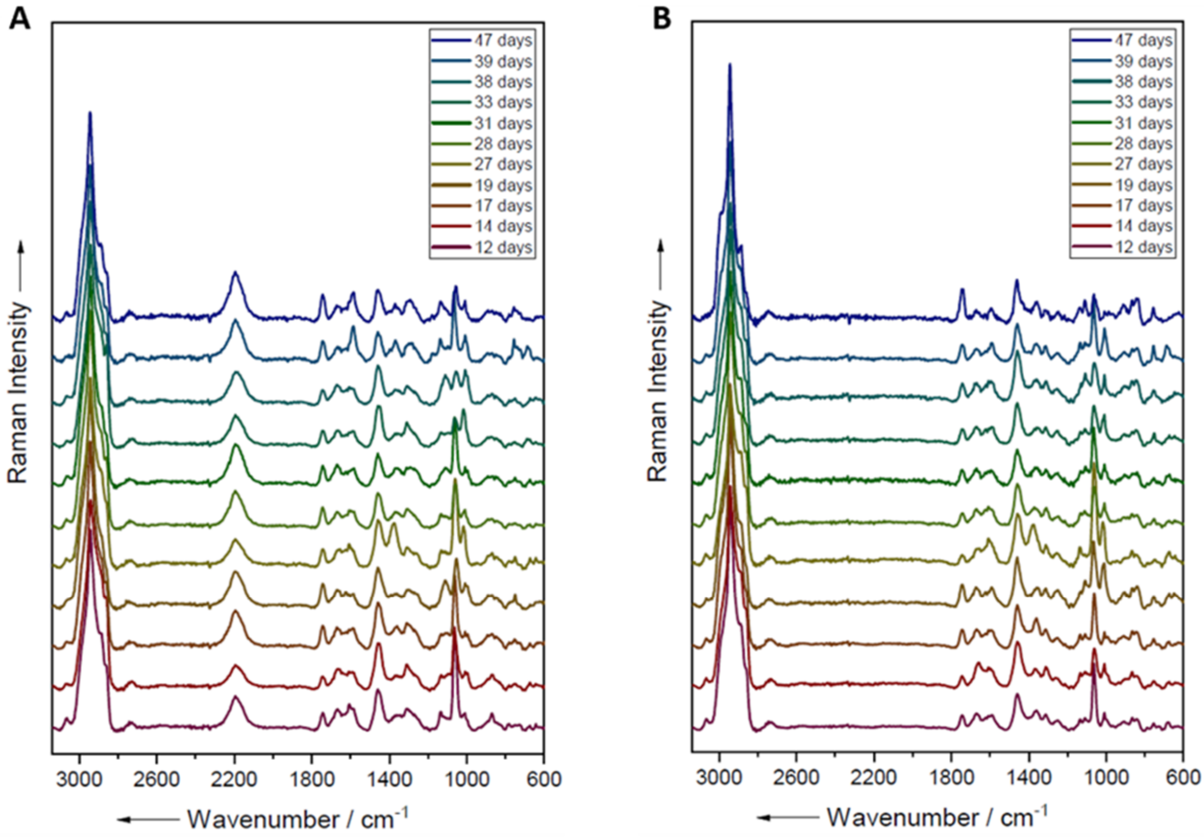

C

	True classes	
**Predicted classes**	**D-labeled**	**Non-labeled**
**D-labeled**	854	112
**Non-labeled**	26	779

Figure S3:

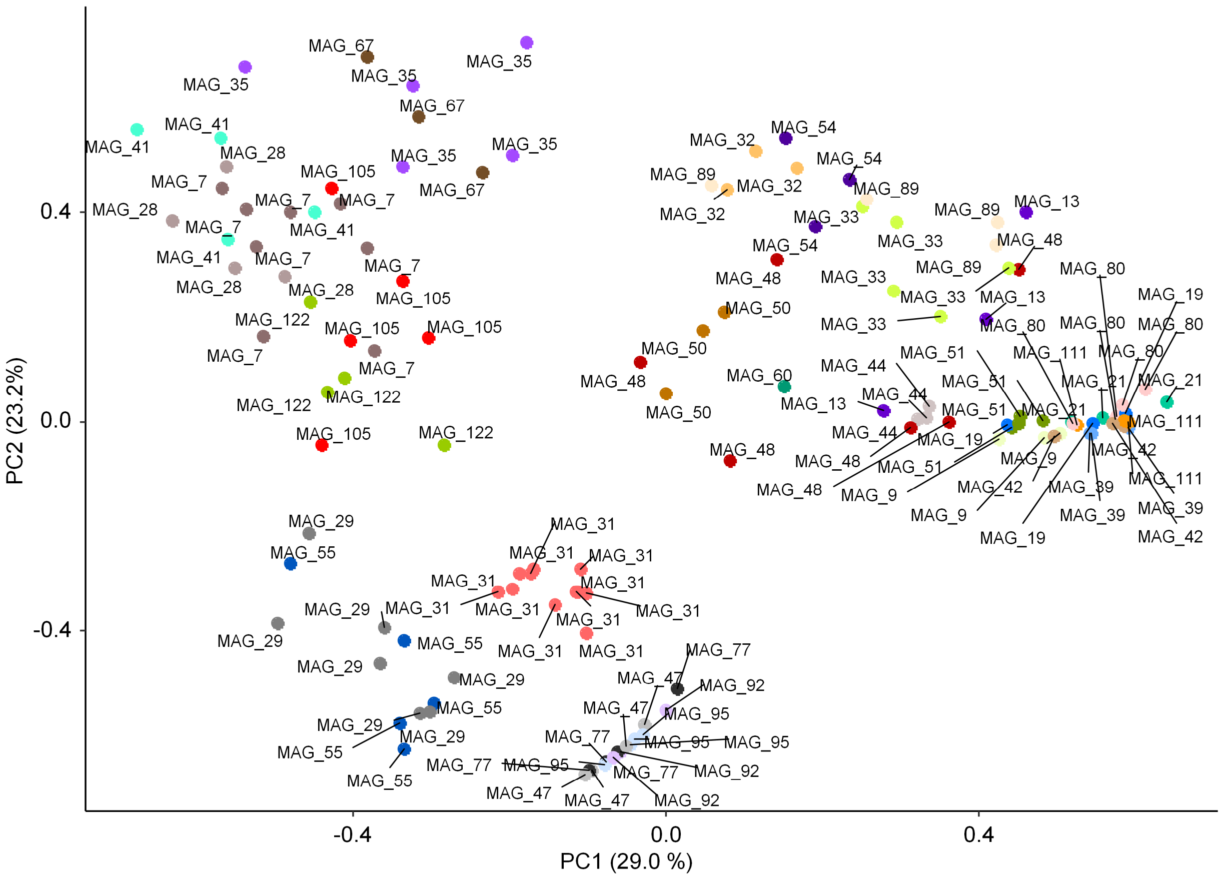

239 **Figure S4:**

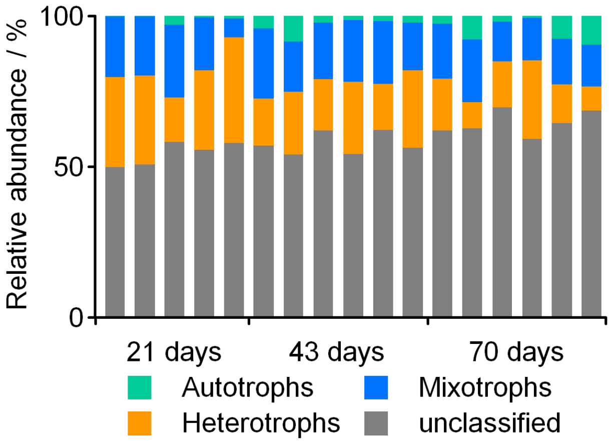

240

241

242 **Figure S5:**

243 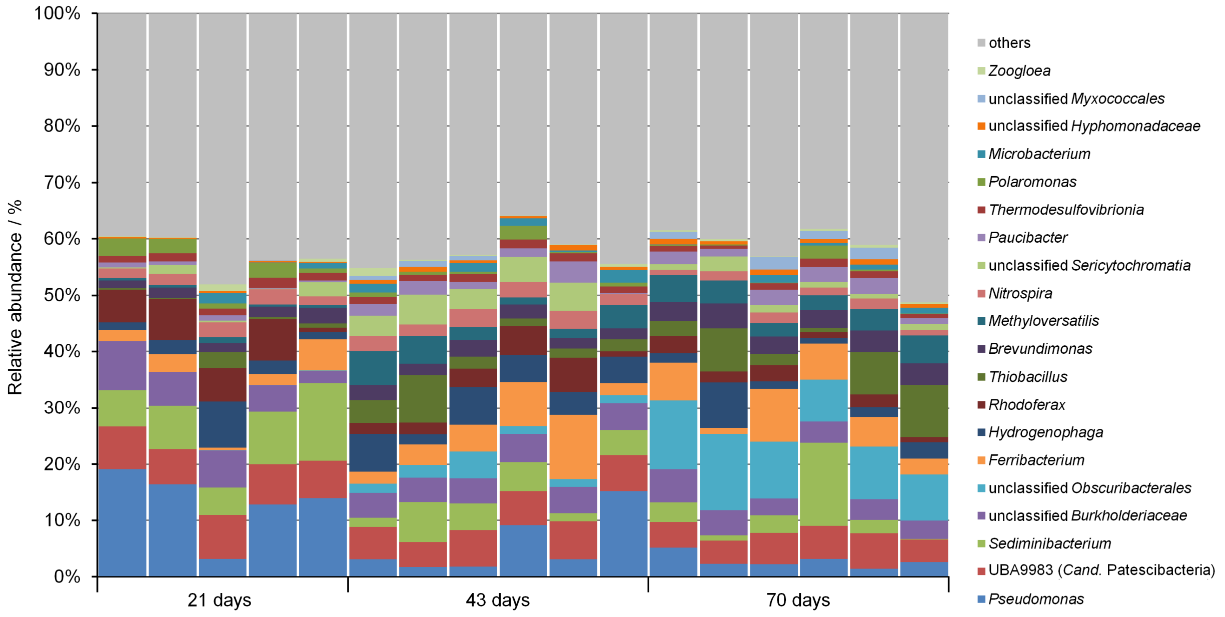

244

Figure S6:

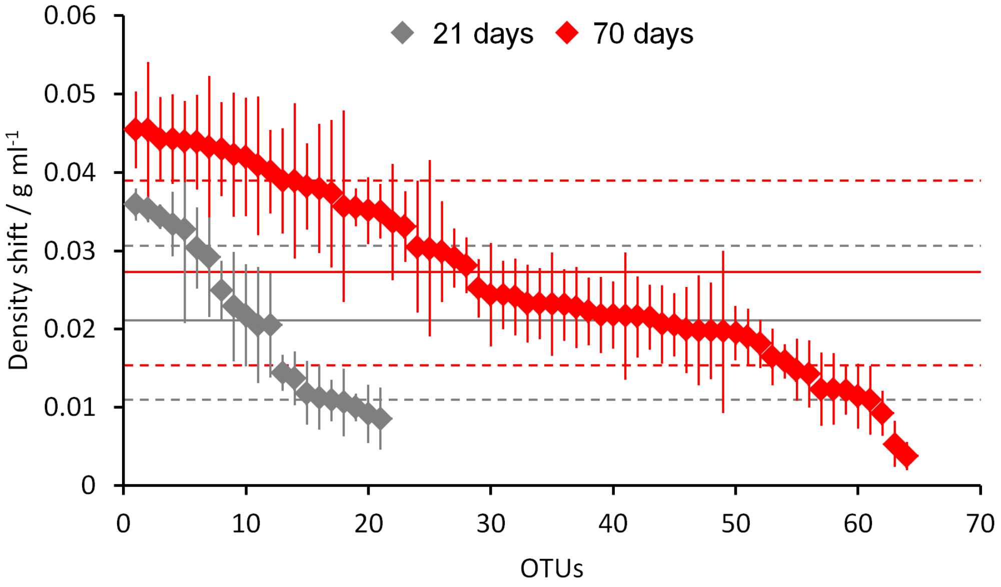
21 days
70 days
Density shift / g ml-1
0.06
0.05
0.04
0.03
0.02
0.01
0
0
10
20
30
40
50
60
70
OTUs

Figure S7:

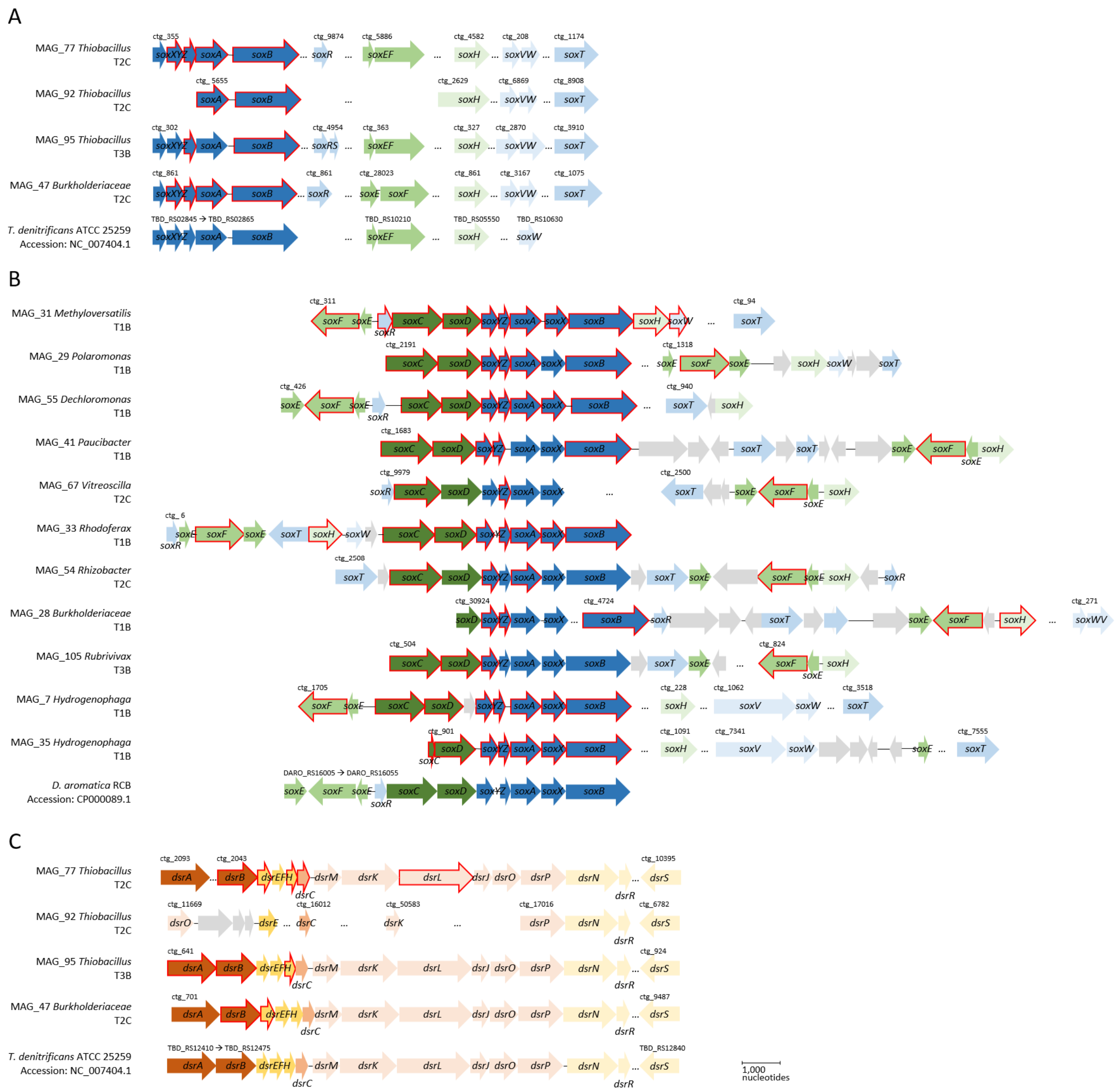
A
MAG_77 *Thiobacillus* T2C
MAG_92 *Thiobacillus* T2C
MAG_95 *Thiobacillus* T3B
MAG_47 *Burkholderiaceae* T2C
T. denitrificans ATCC 25259 Accession: NC_007404.1
B
MAG_31 *Methyloversatilis* T1B
MAG_29 *Polaromonas* T1B
MAG_55 *Dechloromonas* T1B
MAG_41 *Paucibacter* T1B
MAG_67 *Vitreoscilla* T2C
MAG_33 *Rhodoferax* T1B
MAG_54 *Rhizobacter* T2C
MAG_28 *Burkholderiaceae* T1B
MAG_105 *Rubrivivax* T3B
MAG_7 *Hydrogenophaga* T1B
MAG_35 *Hydrogenophaga* T1B
D. aromatica RCB Accession: CP000089.1
C
MAG_77 *Thiobacillus* T2C
MAG_92 *Thiobacillus* T2C
MAG_95 *Thiobacillus* T3B
MAG_47 *Burkholderiaceae* T2C
T. denitrificans ATCC 25259 Accession: NC_007404.1
1,000 nucleotides

Table S1:

BioSample	Genome Accession	Genome name	Taxonomy
SAMN16635724	JADMJG000000000	MAG_13	*Sediminibacterium* sp.
SAMN16635725	JADMJH000000000	MAG_19	*Acidovorax* sp.
SAMN16635726	JADMJI000000000	MAG_21	*Pseudomonas* sp.
SAMN16635727	JADMJJ000000000	MAG_28	Burkholderiaceae bacterium
SAMN16635728	JADMJK000000000	MAG_29	*Polaromonas* sp.
SAMN16635729	JADMJL000000000	MAG_31	*Methyloversatilis discipulorum*
SAMN16635730	JADMJM000000000	MAG_32	Hyphomonadaceae bacterium
SAMN16635731	JADMJN000000000	MAG_33	*Rhodoferax* sp.
SAMN16635732	JADMJO000000000	MAG_35	*Hydrogenophaga* sp.
SAMN16635733	JADMJP000000000	MAG_39	*Rugosibacter* sp.
SAMN16635734	JADMJQ000000000	MAG_41	*Paucibacter* sp.
SAMN16635735	JADMJR000000000	MAG_42	*Acidovorax* sp.
SAMN16635736	JADMJS000000000	MAG_44	Myxococcales bacterium
SAMN16635737	JADMJT000000000	MAG_47	Burkholderiales bacterium
SAMN16635738	JADMJU000000000	MAG_48	*Pseudomonas umsongensis*
SAMN16635739	JADMJV000000000	MAG_50	Eremiobacterota bacterium
SAMN16635740	JADMJW000000000	MAG_51	*Phenylobacterium* sp.
SAMN16635741	JADMJX000000000	MAG_54	*Rhizobacter* sp.
SAMN16635742	JADMJY000000000	MAG_55	*Dechloromonas* sp.
SAMN16635743	JADMJZ000000000	MAG_60	Nitrospirae bacterium
SAMN16635744	JADMKA000000000	MAG_67	*Vitreoscilla* sp.
SAMN16635745	JADMKB000000000	MAG_7	*Hydrogenophaga* sp.
SAMN16635746	JADMKC000000000	MAG_77	*Thiobacillus* sp.
SAMN16635747	JADMKD000000000	MAG_80	*Microbacterium* sp.
SAMN16635748	JADMKE000000000	MAG_89	*Candidatus* Sericytochromatia bacterium
SAMN16635749	JADMKF000000000	MAG_9	*Aquabacterium* sp.
SAMN16635750	JADMKG000000000	MAG_92	*Thiobacillus* sp.
SAMN16635751	JADMKH000000000	MAG_95	*Thiobacillus* sp.
